# Trend in risk of delay in diagnosis of new pulmonary tuberculosis in Northwest China from 2008 to 2017

**DOI:** 10.1186/s12879-019-3725-9

**Published:** 2019-01-30

**Authors:** Hongguang Chen, Tingwei Wang, Lin Liu, Donglin Wang, Qingxue Cheng

**Affiliations:** 1 Key Laboratory of Mental Health, Ministry of Health, Peking University Institute of Mental Health, National Clinical Research Center for Mental Disorders (Peking University Sixth Hospital), Beijing, 100191 China; 2 Yulin Center for Disease Control and Prevention, Yulin, 719000 Shaanxi China

**Keywords:** Diagnostic delay, Pulmonary tuberculosis, Survival analysis

## Abstract

**Background:**

With great changes over the past 10 years in China, especially the rapid economic development, population mobility, urbanization and aging, dynamic change on risk of delay, to our knowledge, has not been well studied in China. The study was to explore risk of delay in diagnosis of new pulmonary tuberculosis (PTB) and dynamic changes in risk of delay in Northwest China.

**Methods:**

From January 1, 2008 to December 31, 2017, a total of 13,603 people with new PTB registered in Yulin city of Shaanxi province were included. The median delay time was estimated by Kaplan-Meier survival curve. Time delay curves of year-, gender-year-, age-year- and smear-year specific were examined using log-rank test. Two-level mixed-effects survival model was used to calculate the hazard ratio (HR) and 95% confidence interval (95%CI) for factors associated with diagnostic delay. Time delay was defined as time interval between the onset of PTB symptoms and being diagnosed. The outcome variable of interest was defined as “being diagnosed” in survival analysis.

**Results:**

The 10-year delay time was 33 days (Interquartile Range, 16–65). Annual median delay time gradually decreased from 60 days to 33 days during the past 10 years. The probability that individuals were diagnosed since onset of PTB symptoms increased by 1.29 times in 2017 when compared to 2008. Female (Hazard Ratio (HR), 95%CI, 0.95(0.91–0.99)), age>45 years (HR, 95%CI, 0.87(0.82–0.93)) and smear positive (HR, 95%CI, 0.86(0.78–0.95)) were associated with increased risk of diagnostic delay over 10-year timespan. However, Age>45 years and smear positive showed trend to be protective factors in the past 5 years.

**Conclusions:**

Time and risk of delay in diagnosis of new PTB had declined over the past 10 years. However, more attentions should be paid to the fact that female still suffered from higher risk of diagnostic delay. We noted a potential reversal in traditional risk factors such as age>45 and smear positive. Those dynamic changes deserved further attention.

## Background

Delay in diagnosis of pulmonary tuberculosis (PTB) results in increasing severity, mortality and transmission. Early diagnosis and immediate initiation of PTB treatment are therefore essential for an effective TB control programme in order to prevent further disease progression at the individual level and transmission within the community [1]. The World Health Organization (WHO) reported that China had made significant progress in TB control and prevention in the past decade, with prevalence being halved and mortality reduced by 78%, suggesting that case detection had improved in China as well [2, 3]). Despite that, annual prevalence (per 100,000 people) declined only minimally from 466 in 2000 to 459 in 2010 [4]. Failure to timely detect PTB played an important role in facilitating the transmission of the disease. This might be linked to the fact that decline in incidence of PTB was less than expected [5]. The main strategies to control TB are early diagnosis and prompt treatment initiation. Passive case-finding is the main approach currently applied by most national TB control programs [1]. However, this passive process has not been as effective as it should be in many low- and middle-income countries [6]. Risk factors associated with delay in diagnosis and treatments of PTB have been identified [3, 5], but dynamic change on risk of delay, to our knowledge, has not been well studied in China.

The first aim of this paper is, therefore, to examine the trend in time of diagnostic delay in Northwest China over a 10-year period from 2008 to 2017. The second is to estimate the dynamic changes of factors associated with diagnostic delay during the last 10 years. We hope that the findings of this study will illustrate the changes that have occurred in Northwest China and provide up-to-date information about potential risk factors affecting diagnostic delay.

## Methods

Yulin city is a prefecture-level city in the Shanbei region of Shaanxi province, China, bordering Inner Mongolia to the north, Shanxi to the east, and Ningxia to the west. It is also one of the cities with rapid economic and social development in economically underdeveloped regions of western China. The city consists of 2 districts and 8 counties with a population of 3.1 million. Every district/county has a dispensary responsible for PTB diagnosis and treatment. From January 1, 2008 to December 31, 2017, all diagnosed PTB patients registered in all 10 dispensaries of Yulin city were included. According to the standard definitions of the Guidelines for Implementing the National Tuberculosis Control Program in China [7], the operating procedures are applied in the diagnosis of PTB.

Smear-positive PTB (PTB+): A patient with two positive direct smear microscopy results, or one positive direct smear microscopy result and one positive sputum culture for PTB, or one positive direct smear microscopy result and radiographic abnormalities consistent with active PTB as determined by a clinician.

Smear-negative PTB (PTB-): A patient with three negative sputum smear results, chest imaging showing lesions of active PTB and one of the following: (a) suspected PTB symptom as cough, expectoration and hemoptysis; (b) strongly positive purified protein derivative (PPD) reaction; (c) positive anti-PTB antibody response; In addition, a patient with positive sputum culture for PTB but negative sputum smear result is also a PTB-case.

Written informed consent forms were obtained from all subjects.

A semi-structured questionnaire was administered to collect the intended data by trained doctors and healthcare workers before the initiation of anti-TB treatment. The information included gender, age, smear test results, onset time of PTB symptoms and time of diagnosis. Diagnostic delay was defined as the duration from onset of any PTB symptom (Chronic cough, Hemoptysis, Night sweat, Fatigue, Fever, Weight loss, Anorexia, Chest pain) noted by the patient to the date of being diagnosed.

### Data analysis and statistics

Survival analysis method is used to analyze data and generally defined as a set of methods for analyzing data where the outcome variable is the time until the occurrence of an event of interest. A nonparametric estimator of the survival function, the Kaplan-Meier method is widely used to estimate and graph survival probabilities as a function of time. A popular regression model for the analysis of survival data is the Cox proportional hazards regression model. It allows testing for differences in survival times of two or more groups of interest, while allowing to adjust for covariates of interest.

The median year-, gender-, age- and smear-specific delay times were estimated by Kaplan-Meier survival curves. Time delay curves of year-, gender-year-, age-year- and smear-year specific were examined using log-rank test. A two-level (level 1 - individuals and level 2 dispensaries) mixed-effects survival model was used to calculate the hazard ratio (HR) and 95% confidence interval (95%CI) for factors associated with delay diagnosis. For the survival analysis in this study, the outcome of interest was defined as “being diagnosed”. Time delay was defined as time interval between the onset of PTB symptoms and being diagnosed. Therefore, Factor with HR less than 1 was regarded as a risk factor associated with an increased risk of diagnosis delay. A two-sided *P* value < 0.05 was considered significant for all analyses. The database was constructed with EpiData v. 3.1 (EpiData Association, Denmark) and data was analyzed using SPSS v. (SPSS Inc., USA).

## Results

### Patient’s characteristics

A total of 13,603 patients with new PTB were included after excluding 381 cases for data incomplete and under age of 15 years. There were 5401 female (39.7% see Table 1) with a median age of 34 and 8202 male (60.3%) with a median age of 39. Male PTB patients were significantly older than female registered from 2008 to 2012 (*P* < 0.05). From 2015 to 2016, female PTB patients instead changed to be older than male (*P* < 0.05). Overall, male PTB patients were older than female over the 10-year timespan (*P* < 0.05). The chi-square test for trend showed that the proportion of male patients increased more than female over the 10 years (Trend χ^2^ = 13.69, *P < 0.001*).

**Table 1:** Age and gender distribution among 13,603 patients with PTB over ten years

Year	Female		Male		*P* ^*^
	N (%)	Median age (IQR)	N (%)	Median age (IQR)	
2008	656 (42.6)	28 (20–54)	884 (57.4)	38 (21–59)	<0.001
2009	682 (39.7)	26 (20–55)	1037 (60.3)	38 (21–59)	<0.001
2010	625 (43.0)	33 (20–59)	829 (57.0)	38 (21–60)	0.046
2011	581 (38.7)	29 (21–56)	920 (61.3)	37 (21–59)	0.056
2012	538 (41.0)	28 (21–55)	775 (59.0)	39 (22–59)	0.001
2013	518 (40.1)	30 (22–59)	773 (59.9)	37 (23–60)	0.131
2014	513 (37.9)	37 (23–60)	841 (62.1)	38 (22–61)	0.609
2015	421 (38.6)	42 (24–63)	669 (61.4)	34 (23–60)	0.002
2016	398 (35.9)	51 (26–67)	711 (64.1)	42 (23–63)	<0.001
2017	469 (38.1)	48 (26–65)	763 (61.9)	49 (26–65)	0.780
Total	5401 (39.7)	34 (21–60)	8202 (60.3)	39 (22–61)	<0.001

Note:”*“Non-parametric Mann–Whitney U test was used to compare annual median age across gender

### Time trends of diagnostic delay from 2008 to 2017

**Fig. 1:**
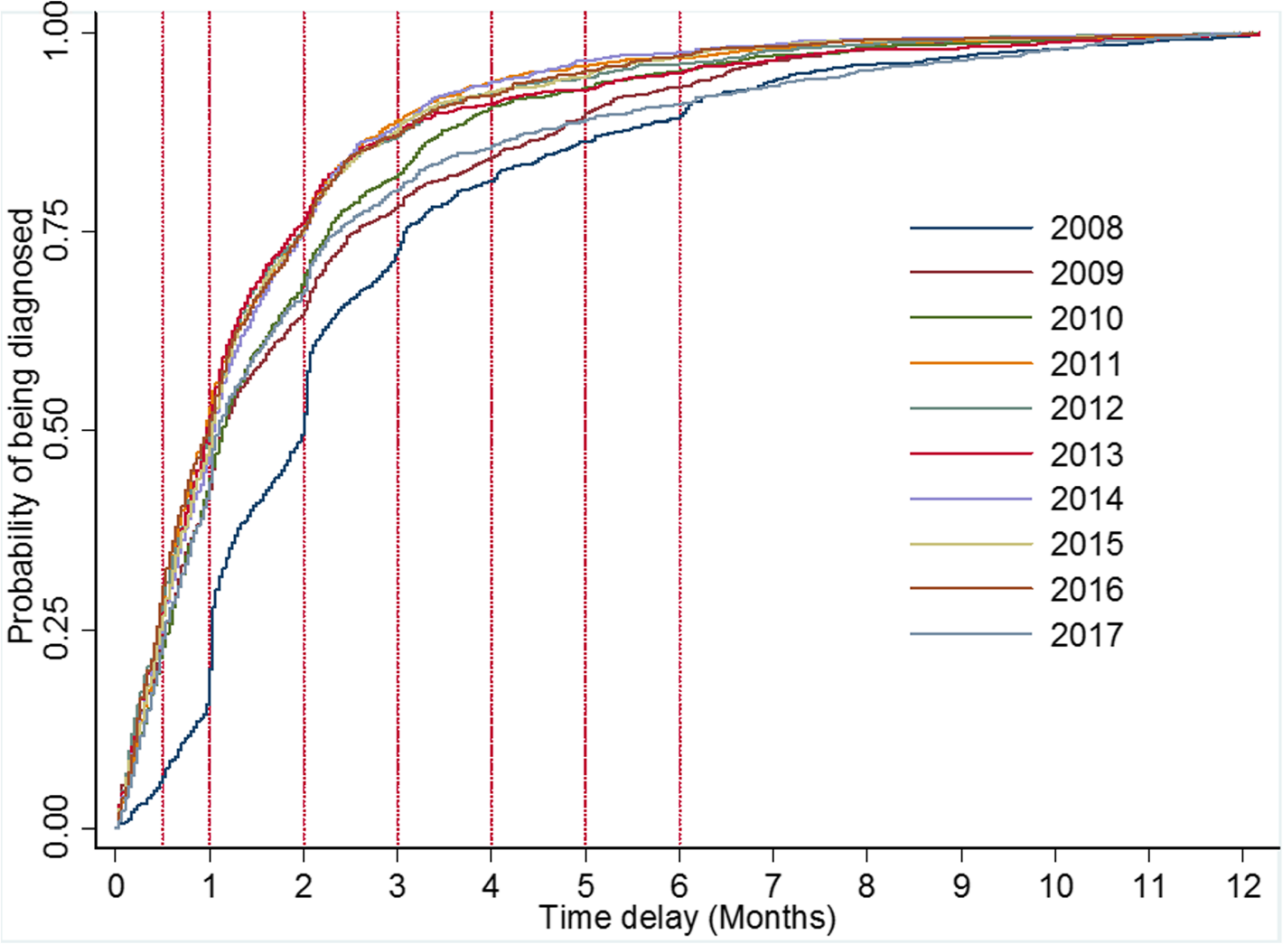
Risk of delay in TB diagnosis from 2008 to 2017

The overall median delay time were 33 days (IQR, 16–65) and annual median delay time gradually shortened from 60 days to 33 days during the past 10 years (see Table 2). The hazard ratios for being diagnosed were also increased gradually compared to that in 2008 in both univariate and multivariable cox proportional hazards regression analysis. A significant difference from the log-rank test showed that year-specific curves for being diagnosed differed (Log-rank test χ^2^ = 365.17, *P* < 0.001, see Fig. 1) and risk for diagnostic delay declined year by year.

**Table 2:** Median delay times and adjusted HRs over ten years

Year	Median delay time (IQR)	Crude HR (95% CI)	*P*	Adjusted HR (95% CI)^*^	*P*
2008	60 (31–93)	1	–	1	–
2009	34 (16–77)	1.31 (1.03–1.67)	0.026	1.31 (1.03–1.66)	0.028
2010	34 (17–68)	1.42 (1.17–1.71)	<0.001	1.41 (1.18–1.69)	<0.001
2011	29 (14–59)	1.71 (1.40–2.08)	<0.001	1.69 (1.39–2.06)	<0.001
2012	30 (13–59)	1.67 (1.36–2.05)	<0.001	1.63 (1.32–2.00)	<0.001
2013	29 (14–57)	1.61 (1.26–2.06)	<0.001	1.55 (1.22–1.97)	<0.001
2014	32 (15–60)	1.68 (1.40–2.03)	<0.001	1.61 (1.34–1.92)	<0.001
2015	31 (14–60)	1.66 (1.37–2.02)	<0.001	1.58 (1.30–1.92)	<0.001
2016	30 (13–59)	1.69 (1.24–2.32)	0.001	1.63 (1.18–2.25)	0.003
2017	33 (16–71)	1.28 (1.01–1.64)	0.044	1.29 (1.02–1.62)	0.030
2008–2017	33 (16–65)	–		–	–

Note: “*” Adjusted for age, sputum smear results, gender. The estimate of variance in level 2 was 0.28 with the standard error of 0.46. An LR test comparing the model with the one-level survival model didn’t favor the random-intercept model with *P* > 0.05

### Variance components estimation each year

LR test comparing the model with the one-level survival regression model favored the random-intercept model with *P* < 0.05 each year from 2008 to 2017 (see Table 3). Therefore, two-level (level 1- individuals and level 2 dispensaries) mixed-effects survival model was used to calculate HR and 95%CI for factors associated with delay diagnosis.

**Table 3:** Estimated variance components each year from 2008 to 2017

Random-effects Parameter	Estimate*	Standard error	95%CI
Dispensaries			
Var (2008)	0.18	0.09	0.07–0.46
Var (2009)	0.37	0.17	0.15–0.92
Var (2010)	0.13	0.06	0.49–0.32
Var (2011)	0.12	0.06	0.04–0.32
Var (2012)	0.12	0.06	0.04–0.33
Var (2013)	0.25	0.12	0.10–0.64
Var (2014)	0.28	0.13	0.11–0.71
Var (2015)	0.05	0.04	0.01–0.20
Var (2016)	0.13	0.07	0.05–0.37
Var (2017)	0.03	0.02	0.01–0.12

Note: “*” LR test with *P* < 0.05 each year from 2008 to 2017

### Time trends of diagnostic delay by gender from 2008 to 2017

**Fig. 2:**
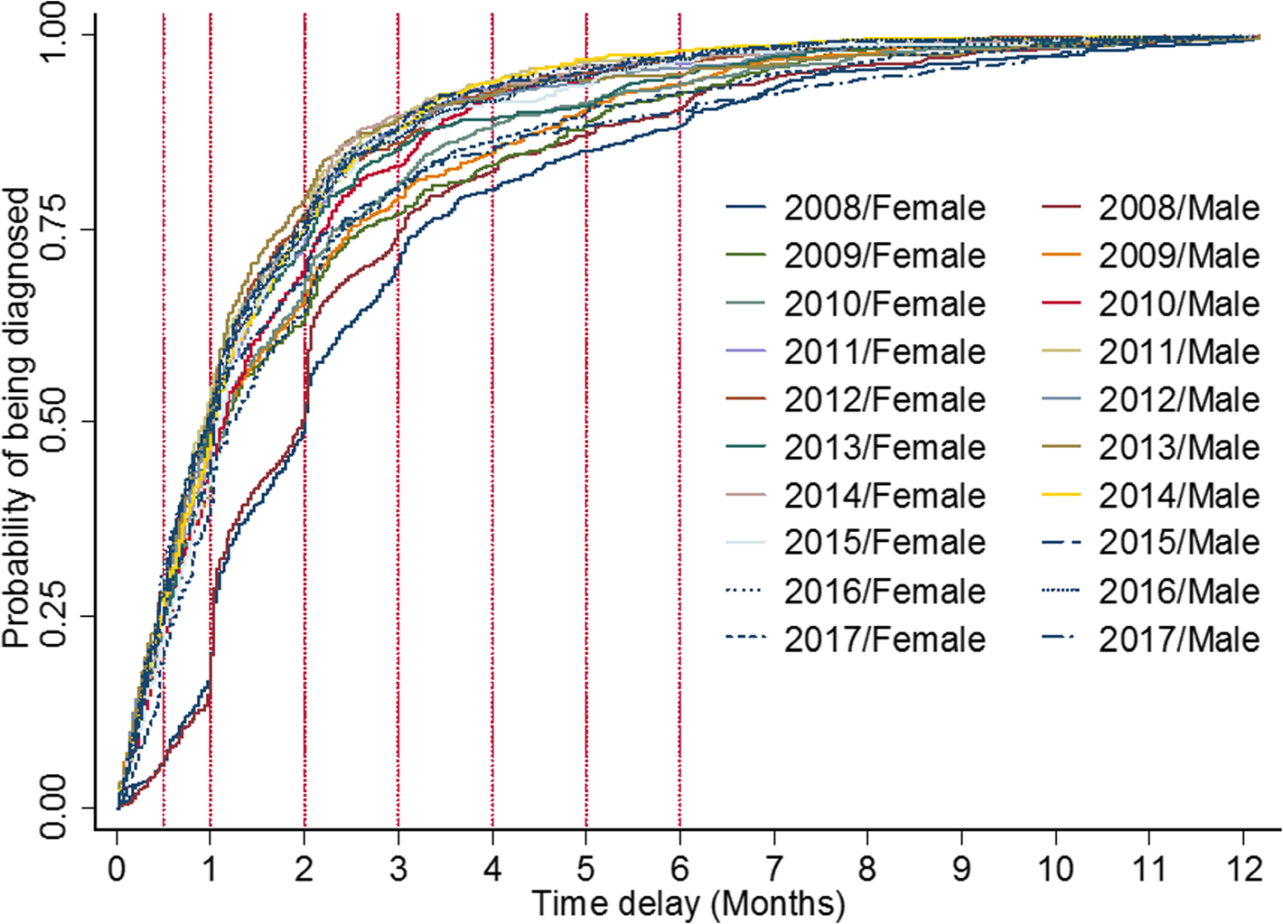
Risk of gender-year-specific delay in TB diagnosis from 2008 to 2017

Overall, median delay in TB diagnosis in female (34 days, see Table 4) is longer than male (32 days). Female (HR, 95%CI, 0.95(0.91–0.99)) was also found to be associated with high risk of delay in PTB diagnosis after adjusting for age, gender, smear results and year. The median delay time in both male and female PTB patients declined during the past 10 years. A significant difference from the log-rank test showed that gender-year-specific curves for being diagnosed differed (Log-rank test χ^2^ = 378.02, *P* < 0.001, see Fig. 2).

**Table 4:** Median delay times stratified by gender and adjusted female-to-male HRs over ten years

Year	Female	Male	Adjusted female: male (HR, 95%CI)	*P*
	Median delay time (IQR)	Median delay time (IQR)		
2008	60 (31–97)	59 (31–91)	0.99 (0.89–1.12)*	0.986
2009	35 (16–79)	34 (16–75)	1.05 (0.95–1.17)*	0.331
2010	34 (17–71)	34 (17–66)	0.94 (0.84–1.04)*	0.221
2011	30 (14–61)	28 (15–57)	0.93 (0.83–1.03)*	0.147
2012	31 (13–57)	30 (14–61)	0.99 (0.88–1.11)*	0.836
2013	30 (16–63)	29 (14–53)	0.91 (0.81–1.02)*	0.099
2014	31 (15–60)	32 (15–60)	0.99 (0.86–1.12)*	0.927
2015	32 (17–61)	29 (13–59)	0.91 (0.80–1.05)*	0.191
2016	29 (13–57)	30 (13–61)	0.96 (0.84–1.10)*	0.570
2017	36 (19–72)	32 (15–69)	0.95 (0.84–1.07)*	0.385
2008–2017	34 (16–67)	32 (15–63)	0.95 (0.91–0.99)†	0.022

Note: “*” Adjusted for age, sputum smear results by using two-level mixed-effects survival model

“†” Adjusted for age, sputum smear results and years. The estimate of variance in level 2 was 0.32 with the standard error of 0.45. An LR test comparing the model with the one-level survival model didn’t favor the random-intercept model with *P* > 0.05

### Time trends of diagnostic delay by age group from 2008 to 2017

**Fig. 3:**
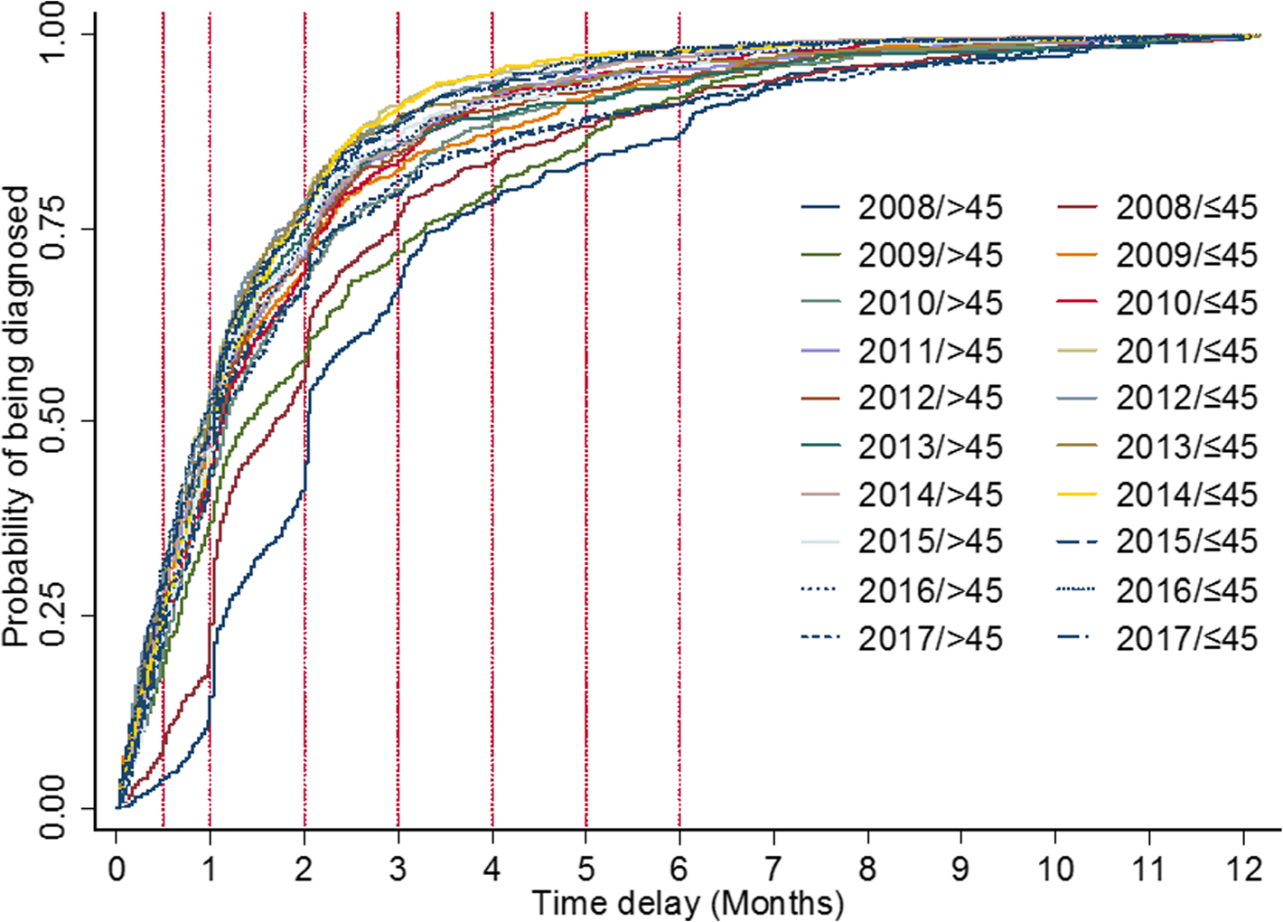
Risk of age-year-specific delay in TB diagnosis from 2008 to 2017

Overall, median delay in age group >45 was 35 days and age group ≤45 was 32 days. However, >45 (HR, 95%CI, 0.87(0.82–0.93), see Table 5) was found to be associated with high risk of diagnostic delay after adjusting for gender, smear results and year. The median delay in both groups presented with decline trend during the past 10 years. A significant difference from the log-rank test showed that age-year-specific curves for being diagnosed differed (Log-rank test, χ^2^ = 439.66 *P* < 0.001, see Fig. 3).

**Table 5:** Median delay times stratified by age and adjusted >45-to- ≤ 45 HRs over ten years

Year	Age>45 years		Age ≤ 45 years		Adjusted >45: ≤45 (HR, 95%CI)	*P*
	N (%)	Median delay time (IQR)	N (%)	Median delay time (IQR)		
2008	526	61 (35–104)	769	51 (31–89)	0.84 (0.75–0.94) ^*^	0.002
2009	644	42 (20–98)	929	32 (14–66)	0.82 (0.74–0.90) ^*^	<0.001
2010	583	35 (18–72)	865	34 (16–66)	0.92 (0.83–1.02) ^*^	0.128
2011	570	30 (15–63)	911	28 (14–56)	0.76 (0.68–0.85) ^*^	<0.001
2012	511	33 (15–65)	783	28 (12–54)	0.74 (0.66–0.83) ^*^	<0.001
2013	511	30 (16–61)	748	29 (13–55)	0.82 (0.73–0.93) ^*^	0.001
2014	524	31 (14–63)	726	32 (15–57)	0.93 (0.82–1.04) ^*^	0.195
2015	390	32 (16–63)	556	31 (13–57)	0.92 (0.80–1.05) ^*^	0.198
2016	485	31 (14–62)	510	27 (12–57)	0.84 (0.73–0.95) ^*^	0.007
2017	617	33 (15–70)	534	32 (17–72)	0.99 (0.88–1.11) ^*^	0.857
2008–2017	5361	35 (17–70)	7331	32 (15–62)	0.87 (0.82–0.93)†	<0.001

Note: “*” Adjusted for gender, sputum smear results by using two-level mixed-effects survival model

“†” Adjusted for gender, sputum smear results and years. The estimate of variance in level 2 was 0.32 with the standard error of 0.45. An LR test comparing the model with the one-level survival model didn’t favor the random-intercept model with *P* > 0.05

### Time trends of diagnostic delay by smear result from 2008 to 2017

**Fig. 4:**
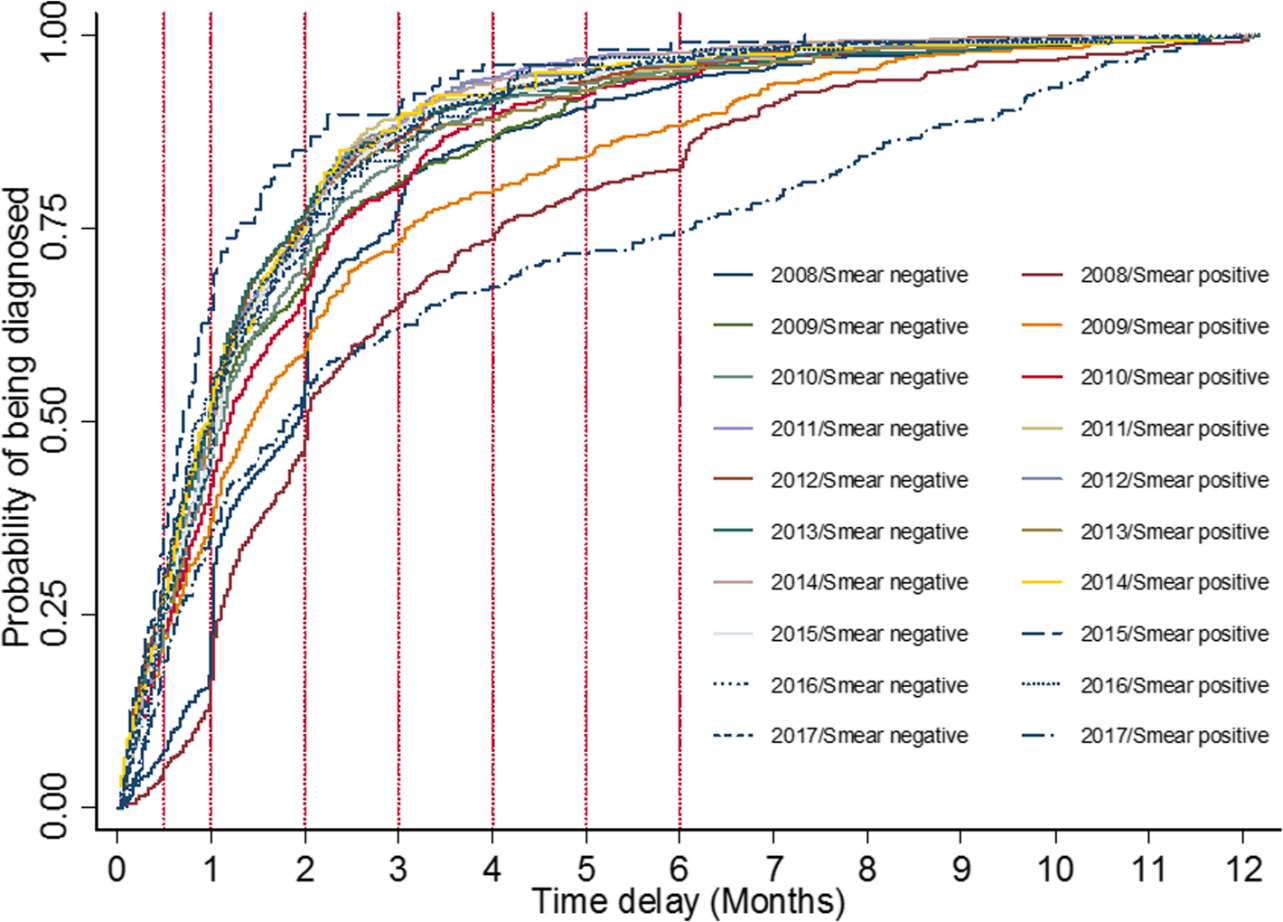
Risk of smear-year-specific delay in TB diagnosis from 2008 to 2017

Overall, median delay in smear positive PTB was longer than smear negative cases. Smear positive (HR, 95%CI, 0.86(0.78–0.95), see Table 6) was found to be associated with higher risk of diagnostic delay after adjusting for gender, age and year. A significant difference from the log-rank test showed that smear-year-specific curves for being diagnosed differed (Log-rank test χ^2^ = 512.73 *P* < 0.001, see Fig. 4). Smear positive was associated with higher risk of diagnostic delay in 2008, 2009 and 2017. However, smear negative presented longer median delay time and showed a shift from a protective factor to be a risk factor in the past 5 years. Even in 2015, this shift ((HR, 95%CI, 1.44(1.17–1.78)) was found to be statistically significant.

**Table 6:** Median delay times stratified by smear results and adjusted Pos-to-Neg HRs over ten years

Year	Smear positive		Smear negative		Adjusted Pos:Neg (HR, 95%CI)	*P*
	N (%)	Median delay time (IQR)	N (%)	Median delay time (IQR)		
2008	518	61 (33–122)	777	58 (31–88)	0.77 (0.69–0.86) ^*^	<0.001
2009	551	43 (17–93)	1022	32 (15–70)	0.83 (0.74–0.93) ^*^	0.001
2010	628	36 (17–70)	820	33 (17–66)	0.92 (0.83–1.02) ^*^	0.124
2011	599	29 (14–59)	882	29 (15–59)	0.98 (0.88–1.09) ^*^	0.742
2012	340	31 (14–60)	954	30 (13–58)	0.97 (0.86–1.11) ^*^	0.693
2013	236	30 (15–61)	1023	29 (14–56)	0.91 (0.78–1.06) ^*^	0.228
2014	169	29 (15–60)	1081	32 (15–60)	1.07 (0.90–1.26) ^*^	0.453
2015	107	21 (12–39)	839	32 (15–62)	1.44 (1.17–1.78) ^*^	0.001
2016	104	25 (14–62)	891	30 (13–59)	0.89 (0.72–1.10) ^*^	0.268
2017	294	53 (20–184)	857	31 (15–62)	0.45 (0.39–0.52) ^*^	<0.001
2008–2017	3546	36 (17–74)	9146	32 (15–62)	0.86 (0.78–0.95)†	0.002

Note: “*” Adjusted for age, gender by using two-level mixed-effects survival model

“†” Adjusted for age, gender and years. The estimate of variance in level 2 was 0.32 with the standard error of 0.45. An LR test comparing the model with the one-level survival model didn’t favor the random-intercept model with *P* > 0.05

## Discussion

This was the first study to explore a 10-year trend in patient delay by using survival analysis in China. Overall, the number of registered PTB cases declined in past 10 years, which complied with overall downloading trend of PTB epidemic in China [8, 9]. However, contrary to the decline trend in the proportion of female cases, male showed rising trend. Male were at higher risk of tuberculosis than female in China because the male-to-female ratio of adults with PTB was about 2:1 or more, reflecting a real risk excess rather than differential detection or notification [10, 11]. The median age of registered PTB cases increased year by year, especially in female cases. This may be linked to the fact that China is rapidly entering into aging society and elderly population is increasing. It is estimated that by 2050 more than a quarter of the population will be over 65 years old [12]. It is believed that as China’s demographic shift to an older society, the epidemic features of tuberculosis will also experience great changes, which deserves further attention [13].

The overall median delay time showed a decline trend from 2008 to 2017. The multivariable cox proportional hazards regression analysis also supported the view that risk for diagnostic delay declined year by year. This was attributed to a series of policies and measures for PTB control and prevention as well as increasing involvement and commitment and public health investment from government over the past 10 years [8, 9].

Female was found to be associated with higher risk of diagnostic delay and also presented longer delay time than male. This was consistent with previous studies conducted in diverse settings [5, 14–16]. Annually, female was significantly associated with diagnostic delay in 2009 and 2013. Whatever, delayed diagnosis in female may lead to more serious clinical, public and social problems and measures to reduce the time delay in PTB diagnosis should be given a high priority [11, 16]. Individuals above 45 years were associated with diagnostic delay in 2008, 2009, 2011, 2012 and 2013 as well as the whole timespan from 2008 to 2017. However, the associations showed no significant difference in the past 3 years. The annual trend predicted that risk for delay in diagnosis was declined among individual above 45 years and increased among individual above 45 years. The phenomena might be linked to urbanization policies and migration and mobility in younger immigrants in recent years [13, 17]. Study from Ethiopia showed that age above 45 years (OR = 2.62, 95% CI, 1.13–6.02) was significantly associated with increased patients’ diagnostic delay [14]. Smear positive was found to be associated with higher risk of diagnostic delay during the timespan from 2007 to 2018. However, the smear positive-to-negative HRs showed inconsistency in recent years (2015 and 2017), possibly due to the decreased variation in risk of delay in diagnosis at the dispensary level (Table 3)**.** After the SARS epidemic was brought under control, the Chinese government implemented a series of measures to strengthen its public health system including increased public health funding, revised laws that concerned the control of infectious diseases, implemented the world’s largest internet-based disease reporting system. Meanwhile, those measures may be generating localized differences in factors affecting diagnostic delay. However, at the national level, this effort coincided with acceleration in efforts to control tuberculosis. Within 3 years, the detection of smear positive cases by public health system more than doubled, from 30% of new cases to 80%. The results also showed a decline trend for the median delay time among smear positive cases. This might also be associated with a new screening strategy among suspects in household contacts of notified tuberculosis cases in China since 2008 [18]. Driven by the new screening strategy, more smear positive PTB patients might be preferentially identified and therefore experience shorter diagnosis delay. In recent years, the proportion of smear negative cases notified is increasing worldwide [9, 19]. The data in this study also showed similar trend from 2008 to 2017. Smear negative cases were likely to experience diagnostic delay for that those cases were often misdiagnosed or missed in their initial visits [20, 21]. Studies reported that smear negative cases appeared responsible for at least one sixth of culture positive episodes of PTB transmission [19]. It was indicated that smear negative was also a major health issue, and probably also a major cause of death [22]. Fortunately, with more DNA-based diagnostic tools developed and to be used in the clinics such as GeneXpert, time delay due to misdiagnosis and missed would be greatly reduced in the foreseeable future [23, 24].

The study included several limitations. Firstly, the definition of the onset of PTB symptom was subjective. As the self-reporting method used in data collection relied on personal recall, this may have led to recall bias. Furthermore, this study had a limited scope to assess the impact of more extensive socioeconomic and cultural factors on diagnostic delay. Additionally, time trends in delay diagnosis might be underestimated for that information on a small number of PTB cases who had not been diagnosed before death was missed.

## Conclusion

It was found that the median delay time was 33 days and showed a decreasing trend over the past 10 years. The probability that people were diagnosed as PTB since onset of symptoms in 2017 increased by 1.29 times when compared to 2008. Female, age above 45 and smear positive were associated with higher risk of diagnostic delay over the 10-year timespan. However, a potential reversal in traditional risk factors such as age>45 and smear positive was noted in the past 5 years, which deserved further investigation before making epidemiological and policy claims.

## Abbreviations

95%CI: 95% Confidence Interval
HR: Hazard Ratio
IQR: InterQuartile Range
LR test: Likelihood-ratio test
PPD: Purified Protein Derivative (PPD)
PTB: Pulmonary Tuberculosis
WHO: World Health Organization
